# BRAIN ACTIVITY DURING DUAL-TASK STANDING IN OLDER ADULTS WITH MILD COGNITIVE IMPAIRMENT

**DOI:** 10.1093/geroni/igac059.2680

**Published:** 2022-12-20

**Authors:** Melike Kahya, Natalia Gouskova, On-Yee Lo, Junhong Zhou, Davide Cappon, Alvaro Pascual-Leone, Lewis Lipsitz, Brad Manor

**Affiliations:** Marcus Institute for Aging Research, Harvard Medical School, Boston, Massachusetts, United States; Hebrew SeniorLife, Roslindale, Massachusetts, United States; Marcus Institute for Aging Research, Boston, Massachusetts, United States; Harvard Medical School/Hebrew SeniorLife, Roslindale, Massachusetts, United States; Marcus Institute for Aging Research, Boston, Massachusetts, United States; Hebrew SeniorLife, Roslindale, Massachusetts, United States; Hebrew SeniorLife, Brookline, Massachusetts, United States; Hebrew SeniorLife/Harvard Medical School, Roslindale, Massachusetts, United States

## Abstract

Performance of a cognitive task while standing disrupts balance in older adults. This disruption is exaggerated in those with mild cognitive impairment (MCI). Moreover, older adults with MCI who exhibit greater dual-task ‘cost’ are more likely to develop falls and dementia. EEG studies suggest that cognitive-motor dual-tasking is associated with brain activity fluctuations originating from central brain regions at specific frequencies, particularly in the alpha-band (8–13 Hz). We hypothesized that older adults with MCI would demonstrate decreased EEG alpha power during dual-task standing compared to healthy controls, and that decreased alpha power would be associated with elevated dual-task cost. We recorded postural sway and EEG in 14 participants with MCI [Montreal Cognitive Assessment (MoCA) < 25] and 16 healthy older adults [MoCA>25] as they completed trials of standing with and without serial subtractions. Postural sway metrics were derived, and from EEG we calculated absolute alpha-, theta-, and beta-band powers within a-priori defined regions-of-interest: the left and right anterior, central, and posterior regions. Repeated Measures ANOVA demonstrated that participants with MCI exhibited decreased alpha power in the central regions during dual-task standing compared to healthy controls (p= 0.01). No significant difference was observed for theta and beta-band powers between participants with MCI and healthy controls. In those with MCI, lower alpha power during dual-task standing correlated with increased dual-task cost to postural sway path (worse balance) (r=-0.4, p=0.03). These results provide preliminary evidence that specific patterns of brain activity during dual-tasking are disrupted in MCI and this is associated with elevated dual-task costs.

